# Effects of Alcohol on Tumor Growth, Metastasis, Immune Response, and Host Survival

**DOI:** 10.35946/arcr.v37.2.14

**Published:** 2015

**Authors:** Gary G. Meadows, Hui Zhang

**Affiliations:** Gary G. Meadows, Ph.D., is the Dorothy O. Kennedy Distinguished Professor of Pharmacy and Hui Zhang, Ph.D., is associate professor in the Department of Pharmaceutical Sciences at Washington State University, Spokane, Washington.

**Keywords:** Alcohol consumption, alcoholism, alcohol use duration, alcohol-induced disease, risk factors, cancer, cancer progression, tumor, metastasis, immune response, immune system, chemotherapy, host survival, angiogenesis, epigenetic mechanisms, treatment, animal models, human studies

## Abstract

Most research involving alcohol and cancer concerns the relationship between alcohol consumption and cancer risk and the mechanisms of carcinogenesis. This review relates the amount and duration of alcohol intake in humans and in animal models of cancer to tumor growth, angiogenesis, invasion, metastasis, immune response, and host survival in specific types and subtypes of cancer. Research on the influence of alcohol drinking on human cancer patients is limited. Although there is more information in animal models of cancer, many aspects still are ill defined. More research is needed to define the mechanisms that underlie the role of alcohol on cancer progression in both animals and humans. Activation of the immune system can play a positive role in keeping cancer under control, but this also can facilitate cancer progression. Additionally, a functional immune system is required for cancer patients to achieve an optimal response to conventional chemotherapy. Insight into the underlying mechanisms of these interactions could lead to effective immunotherapeutic approaches to treat alcoholics with cancer. Defining the epigenetic mechanisms that modulate cancer progression also has great potential for the development of new treatment options not only for treating alcoholics with cancer but also for treating other alcohol-induced diseases.

Alcohol use and abuse have been implicated as etiological factors in the genesis of an increasing number of cancer types in both men and women. In 2012, the International Agency for Research on Cancer (IARC) listed both beverage alcohol (i.e., ethanol) and its major metabolite, acetaldehyde, as tumor-inducing substances (i.e., carcinogens) in humans. The most recent worldwide statistic from 2002 estimated that about 3.6 percent of all cancers, or 389,100 cases, are associated with alcohol consumption (Seitz and Stickel 2007). Cancers for which strong epidemiological evidence indicates that alcohol consumption is associated with an increased risk include, but are not limited to, esophageal, laryngeal, pharyngeal, stomach, colorectal, liver, pancreas, lung, prostate, breast, central nervous system, and skin cancers (Berstad et al. 2008; Boffetta and Hashibe 2006; Brooks and Zakhari 2013; de Menezes et al. 2013; Haas et al. 2012; Kumagai et al. 2013; Longnecker et al. 1995; Nelson et al. 2013; Rota et al. 2014*a*; Watters et al. 2010). The risk of developing a second aerodigestive-tract cancer also is higher in alcohol drinkers (Day et al. 1994; Lin et al. 2005; Saito et al. 2014).

Increased risk of cancer often is associated with high alcohol consumption; however, the specific dose–response relationship varies according to the site of cancer. A recent meta-analysis of 16 articles involving 19 cohorts of subjects with liver cancer (i.e., hepatocellular carcinoma) found a linear relationship between the amount of alcohol consumed and the risk of liver cancer compared with nondrinkers (Turati et al. 2014). Thus, consumption of three alcoholic drinks per day was associated with a moderate increase in risk, whereas consumption of about seven drinks per day was associated with an increase in risk of up to 66 percent. A similar linear relationship has been described for breast cancer risk (Scoccianti et al. 2014).

However, alcohol consumption does not increase the risk of all types of cancer and may even be associated with a lower risk in some cases. For example, although alcohol consumption overall is associated with a higher risk of breast cancer in women, this association does not apply to all types of breast cancer. Thus, among women enrolled in the Women’s Health Initiative the risk of estrogen-positive breast cancer was increased in those who drank alcohol, whereas the risk of triple-negative breast cancer^1^ was reduced among drinkers compared with women who had never consumed alcohol (Kabat et al. 2011).

Interestingly, alcohol consumption also is associated with a lower incidence of several types of blood cancer, including non-Hodgkin’s lymphoma (NHL) (Gapstur et al. 2012; Ji et al. 2014; Morton et al. 2005; Tramacere et al. 2012) and multiple myeloma (Andreotti et al. 2013). An analysis of 420,489 individuals diagnosed with alcohol use disorder (AUD) who were linked to the Swedish Cancer Registry also found a low risk of developing leukemia, multiple myeloma, and Hodgkin’s disease (Ji et al. 2014). Another recent study also showed that alcohol drinking was not associated with increased risk of leukemia and that, in fact, light drinking (less than or equal to one drink per day) was associated with a modest 10 percent reduction in leukemia incidence (Rota et al. 2014*b*). In addition to blood cancers, alcohol consumption also is associated with a lower risk of thyroid cancer (de Menezes et al. 2013) and renal cell carcinoma (Song et al. 2012). In the case of renal cell carcinoma, a lower risk was noted even with consumption as low as one drink per day in both men and women, and higher alcohol intake conferred no further benefit. Finally, a retrospective, observational study of colon and rectum adenocarcinoma indicated that moderate alcohol consumption (less than 14 grams per day) was inversely associated with the incidence of rectal cancer. The investigators also found that moderate intake of beer and especially wine was inversely associated with distal colorectal cancer (Crockett et al. 2011).

In summary, it is well established that alcohol use and abuse is associated with a wide variety of cancers, and the number of these associations continues to grow. At the same time, it now is becoming clear that alcohol can have a preventative effect for certain cancers. Whereas the role of alcohol as a carcinogen is well established, the mechanism(s) by which it prevents cancer are largely unknown and an area for further research. Also, despite the potential beneficial effects of alcohol in the prevention of some cancers, it is important to remember that the detrimental effect of chronic alcohol abuse cannot be disregarded.

Although extensive epidemiologic evidence links the etiology of cancer to alcohol, very little information addresses the critical question of whether and how alcohol modulates tumor metastasis, survival, and the response to cancer therapy. One of the components in these processes is the immune system. Much research regarding the role of the immune response in oncogenesis has centered on hepatocellular cancer (for excellent recent reviews, see Aravalli 2013; Stauffer et al. 2012; Wang 2011). However, less is known regarding the role and interaction among alcohol consumption, immune modulation of tumor growth, blood vessel formation (i.e., angiogenesis), metastasis, and survival. These issues form the major emphasis of this review. It is well established that immunosurveillance by the innate and adaptive immune systems plays important roles in the prevention of cancer and in controlling cancer survival (Fridmann et al. 2012; Rocken 2010). However, direct or indirect interactions of the tumors with their microenvironment can facilitate immune evasion so that the tumor is not detected by the immune system and thus can spread uncontrolled. Tumors also release factors that can directly or indirectly suppress antitumor immune responses, thus facilitating angiogenesis, invasion of surrounding tissues, and metastasis to distant sites in the body (for a general review, see Jung 2011). (For more information on the processes involved in tumor metastasis, see the sidebar.) The following sections will review the role of alcohol in cancer growth and progression, both in humans and in animal models.

## Alcohol, Tumor Growth, and Survival in Humans

### Survival and Mortality

Statistics from 2002 indicate that approximately 3.5 percent of all cancer deaths are associated with alcohol (Seitz and Stickel 2007). A study of 167,343 adult subjects in rural southern India found that daily drinking for 30 or more years increased overall cancer-related mortality (Ramadas et al. 2010). Similarly, a study involving 380,395 men and women who were followed for 12.6 years as part of the European Prospective Investigation into Cancer and nutrition (EPIC) study indicated that compared with no or light-to-moderate consumption (i.e., 0.1 to 4.9 g alcohol/day), heavy (30 or more g/day) drinking in women and heavy to extreme (60 or more g/day) drinking in men was strongly associated with increased total mortality as well as deaths from alcohol-related cancers (Ferrari et al. 2014). However, the effect of alcohol on cancer-specific mortality is variable and depends on factors such as the amount of alcohol consumed, health status of the patient, and the type of cancer.

Survival of patients with oral cavity, pharyngeal, laryngeal, and esophageal cancer is generally reduced by drinking (Jerjes et al. 2012; Mayne et al. 2009; Thrift et al. 2012; Wang et al. 2012*a*; Wu et al. 2012; Zaridze et al. 2009). In Korean patients with head and neck and hepatocellular carcinoma the death rate exhibited a dose-dependent relationship with consumption, with patients who drank between 124 and 289 g of alcohol per day showing the highest death rate (Park et al. 2006). Lower survival of patients with hepatocellular cancer also has been reported in Scotland (Dunbar et al. 2013), Russia (Zarizde et al. 2009), and Spain (Fenoglio et al. 2013). Shortened survival in drinkers as compared with nondrinkers with oral squamous cell carcinoma has been linked to the expression of hypoxia-inducible factor-1-alpha (HIF-1α), a biomarker associated with tumor invasion, metastasis, and progression of a variety of human cancers that also plays a central role in angiogenesis. Drinkers showed higher HIF-1α expression in the nucleus of their cancer cells than nondrinkers (Lin et al. 2008). Finally, although alcohol consumption lowers the incidence of NHL, it decreases patient survival of those with the disease (Battaglioli et al. 2006; Geyer et al. 2010; Talamini et al. 2008).

The effect of alcohol consumption on mortality of women with breast cancer is varied and difficult to interpret. In general, long-term low and moderate alcohol consumption does not seem to affect the survival of breast cancer patients (Flatt et al. 2010; Harris et al. 2012; Kwan et al. 2012; Newcomb et al. 2013). In fact, moderate drinking actually may benefit survival of young women with breast cancer (Barnett et al. 2008; Newcomb et al. 2013). On the other hand, several studies indicated that postmenopausal women with breast cancer who are high-intensity drinkers have lower survival than those with no or lower consumption (Holm et al. 2013; McDonald et al. 2002; Weaver et al. 2013).^2^ In addition to patient age, the specific type of breast cancer may influence the effects of alcohol on survival. Thus, for women with estrogen receptor–positive breast cancer neither pre- nor postdiagnosis alcohol consumption was associated with breast cancer mortality (Ali et al. 2014). In women with estrogen receptor–negative disease, however, mortality was slightly reduced. Another study investigated the effect of pre- and postoperative alcohol consumption over a 3-year period in 934 Swedish primary breast cancer patients who had breast cancer surgery (Simonsson et al. 2014). The study found that both pre- and postoperative consumption of any amount of alcohol was weakly associated with a lower risk of early distant metastases and death. The associations were found in patients with axillary lymph node involvement but not in patients without lymph node involvement.

The effect of alcohol consumption on the incidence as well as the mortality of patients with prostate cancer was evaluated in a prospective cohort study of 194,797 men from the United States aged 50–71 years in 1995–1996 (Watters et al. 2010). The incidence of nonadvanced prostate cancer increased with increasing number of drinks per day, with a 25 percent increase in risk observed after high alcohol consumption (six or more drinks per day). However, an inverse correlation existed between alcohol consumption and deaths from prostate cancer, suggesting that alcohol consumption likely does not affect advanced or fatal prostate cancer.

In summary, several reports indicate that alcohol consumption decreases survival of patients with cancer, whereas other studies did not observe this association. The effect of alcohol consumption on mortality of women with breast cancer is particularly complex and seems to differ according to age, estrogen receptor status, and extent of alcohol drinking. Clearly, more breast cancer–specific studies are needed that correlate mortality with the properties of the cancer and the level of alcohol consumption.

### Tumor Growth and Metastasis

The actual influence of alcohol consumption on tumor growth and metastasis is largely unknown in human cancer patients. Discriminant function analysis of 39 asymptomatic Italian patients with a total of 59 small hepatocellular carcinomas arising from cirrhosis revealed that, among other variables, alcohol intake was a good predictor of tumor doubling time and 2-year survival (Barbara et al. 1992). Another study of 35 Japanese patients with hepatocellular carcinoma and type C cirrhosis found that habitual drinkers consuming 80 g of ethanol per day for 5 years had a statistically significant (*P* < 0.01) shorter tumor-volume doubling time than did non-alcoholic patients (78 ± 47 days vs. 142 ± 60 days) (Matsuhashi et al. 1996).

Basal cell carcinoma—a type of skin cancer—is the most common cancer in humans and continues to increase in incidence. Although the cure rate is high and mortality and morbidity rates are low, aggressive basal cell carcinomas are not rare. In a Spanish study, a significant positive association existed between moderate (5 to 10 drinks per week) and high (more than 10 drinks per week) alcohol consumption and the presence of aggressive basal cell carcinomas (Husein-Elahmed et al. 2012).

## Alcohol, Tumor Growth, Invasion, and Metastasis in Animal Models

Several studies using animal cancer models indicate tumor specific differences in the effect of alcohol on tumor growth and metastasis. These models included various types of breast cancer, melanoma, lung cancer, colon cancer, and hepatocellular carcinoma (For more information, see the sidebar “Effects of Alcohol on Tumor Growth, Invasion, Metastasis, and Survival in Animal Models”). Taken together, these studies and animal models did not allow for general conclusions regarding the impact of alcohol on tumor growth, metastasis formation, and disease progression, as findings differed significantly depending on tumor type. The alcohol model used as well as the duration of alcohol administration also are important variables and can affect the overall outcome (D’Souza El-Guindy et al. 2010), as is the amount of alcohol administered. For example, in studies assessing alcohol’s effects on metastasis formation, acute administration of high doses of alcohol, which mimics binge drinking, generally increased metastasis, whereas longer-term alcohol administration either had no effect or decreased metastasis formation, depending on the amount of alcohol consumed by the animal. Several mechanisms have been suggested as to how acute alcohol may enhance metastasis formation, including alcohol-induced formation of as well as inhibition of various signaling molecules (i.e., cytokines and chemokines). However, although both of these mechanisms seem to contribute to the increase of metastases after acute administration, they do not account for the entirety of alcohol’s effects. Another mechanism whereby alcohol could facilitate metastasis of certain cancers may involve disruption of the integrity of the cells lining the blood vessels (i.e., vascular endothelium). Thus, studies found that exposure to 0.2 percent (weight per volume [w/v]) ethanol in vitro, which promotes angiogenesis and invasion, interferes with the integrity of the vascular endothelium by inducing endocytosis of VE-cadherin (Xu et al. 2012). This molecule is an important component of certain junctions between cells (i.e., cellular adherens junctions). These changes in the vascular endothelium have been shown to allow for increased migration of human A549 lung adenocarcinoma cells, MDA-MB-231 breast cancer cells, and HCT116 colon cancer cells through single-cell layers of endothelial cells (Xu et al. 2012).

**Tumor Metastasis**

Tumor metastasis is the ability of tumor cells to spread from their original site to other sites in the body and to re-establish growth, a new blood supply, and tumor colonies at the new location. **(1)** Cells that escape from a primary solid tumor invade into the surrounding normal tissue by passing through the basement membrane and extracellular matrix (ECM). Several factors are involved in the invasion process, including the ability to activate enzymes called matrix metalloproteinases (MMP), which are important for the tumor cells to degrade basement membranes and underlying stroma. **(2)** The escaped cells reach the blood either directly by actively passing through endothelial cells that line the blood vessels or passively through the lymphatic system, which ultimately carries the tumor cells to the blood. **(3)** Once in the blood, the tumor cells exit into tissues at the secondary site from small capillaries by passing through endothelial cells and then invading the basement membrane of the ECM. **(4)** Once at the secondary site, the tumor cells can lay dormant for extended periods of time, or **(5)** they re-establish growth to form metastatic tumor colonies (by proliferation of cells from a single tumor cell), and finally form a new blood supply (by stimulating the angiogenesis process) to nourish the metastatic tumor. Dormant cells also can proliferate at a future date and ultimately establish a new metastatic tumor. Factors that control the breaking of dormancy are largely unknown, and this is an active area of research.

**Figure:**
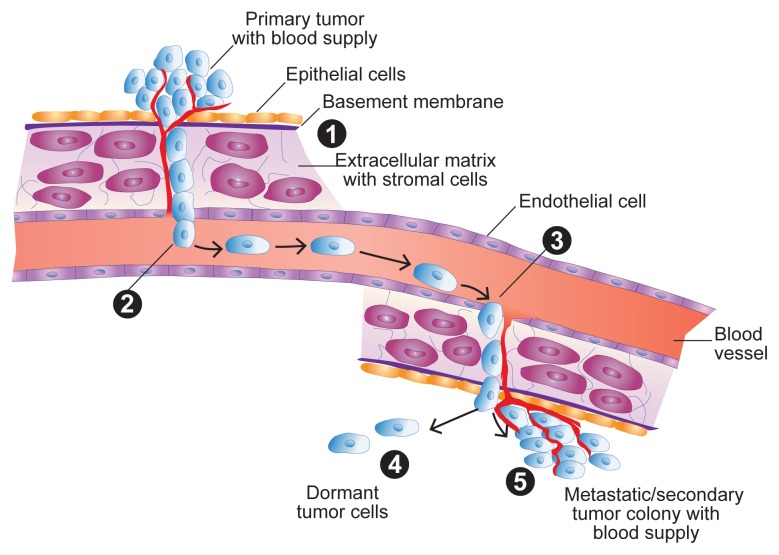

Researchers also examined the effects of alcohol administration on tumor growth. These studies found that high alcohol intake had no consistent effect on tumor growth across different tumors or within a specific tumor type. Low intake of alcohol generally has been associated with enhanced angiogenesis (which promotes tumor growth), whereas high intake may have no effect.

As mentioned earlier, studies in humans found that alcohol’s effects on breast cancer, its progression, and the associated mortality are influenced at least in part by the type of breast cancer involved, specifically its estrogen receptor status. However, animal models involving different breast cancer cell lines detected no consistent trend regarding the effect of alcohol consumption on tumor growth and progression associated with estrogen receptor expression. Estrogen generally suppresses breast cancer growth in vivo but increases in vitro migration of cells away from the original tumor. However, the relationship between estrogen supplementation, diet, caloric intake, and alcohol and their effects on subcutaneous breast cancer growth seem to be highly complex.

The effects of alcohol on in vitro invasion of surrounding tissue primarily have been studied in breast cancer and melanoma cells, with a variety of results. The evidence in melanoma suggests that ethanol can positively impact the extracellular membrane and augment expression of genes that suppress tumor metastasis, resulting in inhibition of metastasis. In addition, certain immune cells called natural killer (NK) cells seem to have some role in regulating the metastasis of breast cancers and melanomas. Clearly, more mechanistic research is needed in murine models to serve as a template for further examination of the complex interactions connecting alcohol to tumor growth, metastasis, and survival in humans.

## Alcohol-Induced Immune Modulation and Tumor Progression

Although many factors influence tumor growth, metastasis, and survival in cancer patients, it is apparent that a functioning immune system plays an important role, not only because it helps control cancer progression but also because it is required for the effectiveness of common cytotoxic chemotherapeutic drugs (Bracci et al. 2014). Evidence that directly implicates immune cells from both the innate and adaptive immune systems in control of cancer growth and progression continues to accumulate. This has stimulated research directed toward developing effective immunotherapeutic approaches to treat cancer (for a review of the tumor immune response as well as approaches being taken to develop immunotherapeutics for cancer, see Harris and Drake 2013).

The innate immune response reacts rapidly to recognize and destroy cancer cells. This response is characterized by inflammatory reactions involving various mediators, including chemokines and cytokines that are produced by a variety of immune cells, such as macrophages, neutrophils, NK cells, and dendritic cells. Macrophages and neutrophils can exhibit antitumor activity as well as suppress immune response against tumor cells (i.e., have immunosuppressive activity). NK cells can destroy tumors on contact, and their antitumor function can be further stimulated by cytokines. Dendritic cells are important in presenting molecules that identify a cell as harmful or foreign (i.e., antigens) to other immune cells and are a bridge between the innate immune response and the B-cell and T-cell responses that characterize the adaptive immune system.

B cells can recognize tumor-cell antigens to ultimately produce antitumor antibodies. They also can have immunosuppressive activity. T cells can be classified according to certain molecules they exhibit on their surfaces, such as CD4, CD8, or CD25. They also can be classified according to their specific functions (e.g., as helper, cytotoxic, regulatory, or memory T cells). CD4^+^ helper T cells can further be divided into Th1, Th2, and Th17 subpopulations based on the specific cytokines they produce and the reactions they induce in the body, which may either facilitate or suppress antitumor immune responses. Certain subsets of CD4^+^CD25^+^ T cells, known as regulatory T cells, generally are immunosuppressive. Cytokines released by Th1 helper T cells, in turn, can activate CD8^+^ T cells, rendering them directly cytotoxic to tumor cells as well as enhance the activity of NK cells. Other populations of CD8^+^ cells (i.e., tumor-specific and memory CD8^+^ T cells) produce high levels of the cytokine interferon gamma (IFN-γ), which is important to the control of tumor metastasis and host survival. Finally, another population of T cells (i.e., NKT cells) that produce a wide variety of cytokines upon activation can function as immunoregulatory cells to either enhance or suppress antitumor immune responses, depending on the cytokine profile that they exhibit. Together, the cells of the immune response provide an intricate interactive control that governs tumor growth and progression. (For more information on the innate and adaptive immune systems and their responses, see the “Primer on the Immune Response,” by Spiering.)

### A Role for the Immune System in Control of Cancer Progression

Numerous findings with a variety of tumor types suggest that the numerous types of immune cells, particularly various T-cell subpopulations, are involved in controlling tumor progression, including the following:

- CD8^+^ T cells, in particular a subtype expressing the memory phenotype (CD8^+^CD44^hi^) that produce high levels of IFN-γ, are key to controlling metastasis and host survival of different tumors (Erdag et al. 2012; Eyles et al. 2010; Fridman et al. 2012; Rosenberg and Dudley 2009).
- Increased tumor progression in patients with gastric cancer has been tied to increased peripheral blood levels of certain CD4^+^ T-cell subpopulations, including Th22 (CD4^+^IL-22^+^IL-17^−^IFN-γ^−^) and Th17 (CD4^+^IL-17^+^IFN-γ^−^) cells (Liu et al. 2012).
- A multivariate analysis in metastatic breast cancer patients indicated that prolonged progression-free survival was correlated with increased CD3^+^CD4^+^ or CD8^+^CD28^+^ T cells. Conversely, elevated CD8^+^ CD28^−^ T cells were associated with shortened progression-free survival (Song et al. 2013). These effects seem to be related to the cytokines produced by these cells, because patients with elevated CD8^+^CD28^−^ and CD4^+^CD25^+^ T cells had elevated levels of IL-6, and the patients that expressed elevated CD8^+^CD28^−^ T cells also exhibited decreased IFN-γ.

These data underscore the importance of immune cells in the progression of cancer.

Alcohol can modulate the body’s immune responses, and it is possible that these alterations affect disease progression in cancer patients. For example, in a Chinese study of newly diagnosed NHL patients (Lin et al. 2009), alcohol addiction was associated with increased peripheral blood CD4^+^ CD25^hi^CD127(IL-7)^lo^ regulatory T cells, and these increases were higher in male than in female patients. However, the increased levels of these cells did not relate to the clinical features (e.g., age, tumor staging, cancer symptoms, pathological subtype, and short-term treatment efficacy). Therefore, the importance and significance of the elevated regulatory T cells is uncertain in NHL.

Another study of 25 patients with hepatocellular carcinoma in Japan (Yang et al. 2006) found an increase in CD4^+^CD25^+^ T cells in the tissue regions surrounding the tumor (i.e., the peritumoral region) compared with similar tissues in patients who had chronic hepatitis or liver cirrhosis but no hepatocellular carcinoma. The values were not correlated with the stage of the tumor.^3^ These peritumoral CD4^+^CD25^+^ T cells had a regulatory phenotype, as indicated by an increased expression of several molecules (e.g., cytotoxic T lymphocyte antigen 4 [CTLA-4, CD152] and glucocorticoid-induced TNF receptor superfamily member 18 [GITR, CD357]), expression of a biomarker for regulatory T cells (i.e., FOXP3), and decreased expression of CD45RA. The numbers of these cells were inversely associated with the numbers of CD8^+^ T cells. Additional observations suggest that these regulatory T cells may contribute to the progression of hepatocellular carcinoma by interfering with normal immune responses. Thus, isolated peritumor CD4^+^CD25^+^ T cells that were incubated with peripheral blood T cells from the same person and stimulated with certain antibodies, suppressed T-cell proliferation and activation of CD8^+^ T cells (Yang et al. 2006).

The functionality of the innate immune system also can be correlated with tumor progression. A recent study compared innate immune-system functionality with the number of circulating tumor cells in patients with a variety of cancers. In patients with metastatic disease, these circulating tumor cells are promising as biomarkers for tumor progression and overall cancer survival, with relatively high circulating cell numbers correlated with a poor prognosis. The study, which included patients with metastatic breast, colorectal, and prostate cancer found decreased NK cell cytolytic activity and decreased expression of certain proteins (i.e., toll-like receptors 2 and 4) in patients with high circulating tumor cells compared with patients with relatively low numbers (Santos et al. 2014). Decreased NK cytolytic activity also has been linked with other types of cancer, including colorectal cancer (Kim et al. 2013), metastatic melanoma (Konjevic et al. 2007), and head and neck cancer (Baskic et al. 2013).

In addition to the effects of specific types of lymphocytes on cancer growth and metastasis, chemokines also have important roles in cancer progression, terminal growth arrest of tumor cells (i.e., tumor growth senescence), angiogenesis, epithelial mesenchymal transition,^4^ metastasis, and evasion of the immune system. Chemokines and their receptors often are altered in cancer patients, and their importance in cancer progression has been the subject of several recent reviews (Aldinucci and Colombatti 2014; de Oliveira et al. 2014; Sarvaiya et al. 2013).

### Alcohol and Immune Effects in Patients with Cancer

A large body of literature indicates that alcohol consumption modulates many aspects of the innate and adaptive immune systems. Alcohol originally was described as immunosuppressive, and numerous studies support the immunosuppressive aspects of alcohol consumption on the innate and adaptive immune systems. However, it also is well documented that chronic alcohol administration can activate the immune system—especially dendritic cells, T cells, and NKT cells—in experimental animals as well as humans (Cook et al. 1991; Laso et al. 2007; Song et al. 2002; Zhang and Meadows 2005). This adds to the complexity of interpreting alcohol’s effect on cancer progression and survival.

Few studies have specifically examined the interaction between alcohol and the immune response in cancer patients or in experimental animals implanted with cancer cells. Although human cancer patients often have immune deficits, few data are available that specifically address the effects of alcohol on immune parameters. The studies that are available examined the immune responses in patients with head and neck cancer. These patients often are immunodeficient because of their alcohol abuse and heavy tobacco use; however, the contribution of continued alcohol abuse to altered immune parameters in these patients has largely not been assessed.

An early study of patients with head and neck squamous cell carcinoma and a history of smoking and significant alcohol use found a deficiency in the percentage of certain T cells (i.e., Th5.2^+^ IL-2–producing T cells) in peripheral blood compared with control patients who were hospitalized for elective surgical procedures (Dawson et al. 1985). The overall percentage of all T cells, as well as of CD4^+^ T-, CD8^+^ T-, B-, and NK cells, in contrast, did not differ between cancer and control patients. However, this effect cannot be clearly attributed to alcohol because the patients also were heavy tobacco users. Another study compared a different indicator of immune-system function (i.e., production of antigen-specific antibodies) using blood samples obtained from patients with squamous cell carcinoma of the oropharynx or larynx and healthy controls, some of whom had high alcohol consumption (i.e., 100 g/day) and/or excessive smoking (20 cigarettes per day for more than 5 years) (Wustrow 1991). The study found that among healthy participants, those with high alcohol consumption or smoking had a pronounced decrease of antigen-specific antibody production in vitro. The effect was more pronounced in heavy drinkers than in excessive cigarette smokers. Cancer patients who were heavy drinkers, in contrast, did not show any antigen-specific antibody production in vitro. However, after removal of a subset of white blood cells (i.e., mononuclear cells) from the peripheral blood, samples from two-thirds of the patients began to produce such antibodies, and antibody production reached the same level as that measured in the healthy subjects with high alcohol abuse and cigarette consumption. The author suggested that the decreased antigen-specific antibody production in the cancer patients could be related to upregulation of suppressive cells in these patients (Wustrow 1991).

More recent studies have evaluated the role of a protein called macrophage migration inhibitory factor (MIF), which is an important regulator of the innate immune response. This factor has been studied in patients with lip or intra-oral squamous carcinoma as well as in patients who consumed alcohol regularly (Franca et al. 2013). The analyses found a significant relationship between the incidence of intra-oral cancer, alcohol use, and the number of MIF-positive cells in the stroma. Thus, MIF in the stroma of intra-oral tumors (i.e., tongue, floor of mouth, and alveolar ridge) was decreased in patients who consumed alcohol. The importance of these findings is unknown, although patients with tumors that did not express MIF had a worse prognosis than patients that did.

### Alcohol and Immune Interactions in Animal Models of Cancer

If human tumor cells are introduced (i.e., inoculated) into animals with functioning immune systems, they do not form tumors because they are recognized as foreign by the animal’s immune system. However, human tumors often grow in animals with compromised immune systems, and such animals can be used as models for a variety of research questions, including studies regarding the roles of various immune cells in controlling cancer and the impact of alcohol on this process. One such study specifically examined the role of CD4^+^ T cells in regulating tumor growth by implanting cells from a human lung cancer (i.e., the 201T human lung adenocarcinoma cell line) into the lungs of a strain of mice called BALB/c (Hunt et al. 2000). In this study, the mice were administered alcohol chronically for 8 weeks and then were injected with an anti-CD4 monoclonal antibody to deplete CD4^+^ T cells. Initial experiments confirmed that normal, immunocompetent BALB/c mice did not form lung tumors. To examine the effect of alcohol, the mice were administered ethanol in their food^5^ as well as 10 percent in their drinking water throughout the experimental period. After 8 weeks of ethanol administration or regular food, the mice were implanted with the tumor cells and also received one injection of the anti-CD4 antibody. Separate groups of mice were evaluated at 6 weeks and 13 weeks. Mice in the non–ethanol-fed control group injected with one dose of anti-CD4 antibody initially developed large tumors at 6 weeks, which significantly regressed thereafter. Compared with these control animals, the ethanol-fed mice exhibited significantly larger tumors at 6 weeks as well as a diminished ability to decrease their tumor size at 13 weeks. The findings suggest that this difference in the ability of the ethanol-fed mice to reduce their tumor burden results from an impaired immune system caused by chronic alcohol intake.

Another series of studies analyzed the interaction between chronic alcohol consumption and immune-system functioning in female C57BL/6 mice implanted with B16BL6 melanoma cells under the skin (i.e., subcutaneously). In these studies, the animals continuously received 20 percent w/v ethanol in the drinking water and generally were inoculated with B16BL6 melanoma after 12 weeks or longer of this treatment. The analyses found that in the alcohol-exposed, melanoma-bearing animals the overall numbers of peripheral blood lymphocytes (which include various types of immune cells) were lower than in water-drinking controls when determined 11, 14, and 17 days after tumor inoculation (Zhang et al. 2012). This was in contrast to normal mice not injected with melanoma cells, in which the number of lymphocytes was not altered by alcohol. The decrease in cells was not caused by cell death (i.e., apoptosis). Additional analyses demonstrated that the lowered lymphocyte numbers (i.e., lymphopenia) were associated with a two- to fourfold decrease in mature B cells as well as in CD4^+^ and CD8^+^ T cells. Further examination demonstrated that the decrease in mature B cells in the blood was associated with impaired B-cell circulation resulting from a down regulation in the formation of compound called sphingosine-1-phosphate and its receptors. Formation of sphingosine-1-phosphate is mediated (i.e., catalyzed) by an enzyme called sphingosine kinase 1, which is an important regulator of tumor progression in melanoma and several other cancers (Meng et al. 2014). This enzyme and other components of the sphingosine-1-phosphate pathway currently are being examined as potential targets for cancer drug development (Pyne and Pyne 2013; Tabasinezhad et al. 2013). Zhang and colleagues (2012) concluded that the severe decrease in mature B cells in the blood of the alcohol-exposed and tumor-inoculated animals could result from inhibition of B-cell migration from the spleen to the blood resulting from impairment of the sphingosine-1-phosphate signaling pathway. The importance and role of mature B cells in antitumor immune responses is still unclear. They play a dual role by both inhibiting (Inoue et al. 2006) and facilitating antitumor immune response through production of cytokines and enhancement of T-cell activation (DiLillo et al. 2010). Thus, impaired circulation of B cells attributed to alcohol consumption (Zhang et al. 2012) could negatively affect T-cell function.

The investigators also analyzed the levels of the various types of blood cells in the spleen (Zhang et al. 2012). The spleen contains proportionally more B cells and fewer T cells than the peripheral blood; among the T cells, the spleen normally contains a higher proportion of CD8^+^ T cells than the peripheral blood. The analyses found that alcohol consumption also led to a decrease in CD8^+^ T cells in the spleen; however, this reduction was less remarkable than in peripheral blood. No changes in these cells were observed in the bone marrow. Furthermore, alcohol consumption reduced the overall numbers of B cells in the spleen, although it did not affect all types of B cells equally. Thus, there was no effect on splenic follicular B cells, whereas the number of immature T1 B (CD19^+^CD93^+^CD23^−^) cells increased and the number of marginal zone B cells (CD19^+^CD1d^hi^CD21^hi^) decreased.

**Glossary**

Antibody: Immune molecule (protein) produced by *B cells* that recognizes foreign molecules that have entered the body (i.e., *antigens*), binds to these molecules, and marks them for destruction by the body’s immune system
Antigen: Any molecule that can bind specifically to an *antibody* and can induce an immune response
B cells: One of the two main types of lymphocytes involved in the adaptive immune response; when activated by interacting with a specific *antigen*, they differentiate into specific subtypes and begin to produce *antibodies* that recognize the specific *antigen*
Chemokines: Small proteins that serve as chemoattractants, stimulating the migration and activation of cells, particularly phagocytic cells and lymphocytes; they have a central role in inflammatory responses
Cytokine: Any of a group of molecules, produced primarily by immune cells, that regulate cellular interactions and other functions; many cytokines play important roles in initiating and regulating inflammatory reactions
Dendritic cell: A type of immune cell involved in the innate immune response that is characterized by a branched morphology; dendritic cells can bind to *antigens* and present these antigens to *T cells,* thereby initiating an adaptive immune response
Macrophage: A type of immune cell that ingests foreign particles and micro-organisms in a process called phagocytosis and which synthesizes *cytokines* and other molecules involved in inflammatory reactions
Natural killer (NK) cell: A type of immune cell involved in the innate immune response that can kill certain harmful cells, particularly tumor cells, and contributes to the innate immune response to cells infected with viruses or other intracellular pathogens
Neutrophil: A type of immune cell involved in the innate immune response that engulfs and kills extracellular pathogens in a process called phagocytosis
T cells: One of the two main types of lymphocytes involved in the adaptive immune response after activation through the interaction with a specific *antigen*. T cells can be divided into several subgroups that support other immune cells (helper T cells), kill invading pathogens or infected cells (cytotoxic T cells), or help turn off the adaptive immune response (regulatory T cells)

Other analyses (Zhang and Meadows 2010) investigated the effects of chronic alcohol consumption on various types of CD8^+^ T cells in mice with or without inoculation of B16BL6 melanoma cells. These analyses yielded the following results:

- CD8^+^CD44^hi^ T memory cells produced high levels of IFN-γ and were important in the antitumor response to B16BL6 melanoma. Mice not inoculated with melanoma that chronically consumed alcohol had higher levels of these memory cells than mice that drank water.
- After melanoma inoculation, these CD8^+^CD44^hi^ T memory cells increased over a 2-week period in water-drinking animals. However, in mice that chronically consumed alcohol, these memory cells failed to expand in response to melanoma inoculation.
- The lack of expansion of the memory T cells in response to melanoma inoculation in the alcohol-consuming mice resulted from a reduced ability of these cells to proliferate in response to melanoma. Additional experiments examined the ability of CD8^+^ T cells obtained from 2-week melanoma-bearing mice to proliferate in vitro in response to specific T cell stimulation (i.e., anti-CD3 and anti-CD28 anti-bodies). The analyses showed that proliferation of CD8^+^ T cells was reduced by more than one-half in alcohol-consuming mice compared with cells from water-drinking mice.
- The number of CD8^+^ T cells that specifically recognize a melanoma-specific antigen (i.e., gp100) was 2.5-fold lower in the spleen of the alcohol-consuming mice than in water-drinking control mice at three weeks after tumor inoculation, suggesting an impaired immune response.
- The percentage of IFN-γ–producing CD8^+^ T cells, which have tumor-suppressive effects, initially displayed a robust increase until day 11 after melanoma inoculation, but exhibited an accelerated decay thereafter, suggesting enhanced inhibition of these cells related to an alcohol–melanoma interaction.

The investigators also analyzed the numbers of several types of cells whose production is induced by tumors and which produce factors that inhibit the antitumor functions of T cells, including myeloid-derived suppressor cells (MDSCs), tumor-associated macrophages, T regulatory cells (CD4^+^CD 25^+^FOXP3^+^), regulatory B cells (CD1d^hi^CD5^+^), and NKT cells (Zhang and Meadows 2010; Zhang et al. 2012). Of these, the percentage of CD11b^+^Gr-1^int^ MDSCs, as well as the percentage of the CD124^+^ subpopulation within the CD11b^+^Gr-1^int^ cells, was increased in the peripheral blood of alcohol-consuming mice as determined one week after tumor inoculation. These cells are known to suppress antitumor T-cell immune responses (Sinha et al. 2005; Terabe et al. 2005; Zhu et al. 2007). The percentages of T regulatory cells and tumor-associated macrophages did not differ between alcohol-consuming and water-drinking mice with melanoma tumors (Zhang and Meadows 2010).

The percentage and number of CD3^+^NK1.1^+^ invariant NKT cells was elevated in the blood of alcohol-consuming, B16BL6 melanoma-bearing mice especially at day 14 after tumor inoculation (Zhang et al. 2012). These cells have important regulatory functions and can either promote antitumor immune responses or inhibit them. Initially, these cells express a cytokine profile that favors antitumor immune responses (i.e., a high ratio of IFN-γ to IL-4). After repeated activation, however, these cells become anergic and switch to a cytokine profile that inhibits anti-tumor immune responses and favors tumor progression (i.e., a high ratio of IL-4 to IFN-γ) (Parekh et al. 2005). The invariant NKT cells from the alcohol-consuming, melanoma-bearing mice exhibit a high IL4/IFN-γ ratio, indicating that they express a cytokine profile favoring immune inhibition and tumor progression (Zhang et al. 2015).

Overall, very few studies have addressed the role of and interaction among alcohol, cancer, and the immune system once the cancer is established. It is important to understand these interactions, however, because many alcoholics have immune deficiencies and because a competent immune system is important to the success of many conventional drug therapies for cancer. In addition, new immune-enhancing approaches to cancer therapy are being developed. Finally, evidence from animal models and human studies suggests that appropriately combined chemotherapy and immunotherapy may be more beneficial than either therapeutic approach alone (Ardiani et al. 2013; Shi et al. 2014; van Meir et al. 2014; Wang et al. 2014).

## Additional Avenues for Future Research

The interactions between alcohol use/abuse, the antitumor immune response, tumor growth, and spread of cancer are complex. A negative impact of alcohol on the immune system can lead to increased cancer mortality; however, studies also indicate that alcohol, generally in low doses, can have beneficial effects on mortality, depending on the cancer. Clearly, more mechanistic research is needed to define the complex interactions between cancer and alcohol. Additional research is likely to uncover targets to mitigate the detrimental effects of alcohol on mortality and to identify specific biochemical and molecular mechanisms involved in the beneficial effects of alcohol related to enhancing survival of cancer patients. This research could translate into the development of more effective and specific targeted approaches to treat cancer patients in general and especially those who abuse alcohol.

Because cancer is a collection of different diseases with diverse underlying causes, it is important that research take into account the diversity in gene mutations and alterations involved in uncontrolled growth. In addition, future analyses must address the genetic instability that fosters metastasis, the major cause of death from cancer. It is becoming increasingly clear that genes which suppress metastasis (Meadows 2012) as well as signaling pathways that inhibit metastasis (Singh et al. 2014) can be regulated through epigenetic mechanisms^6^ induced by the diet and dietary constituents, including alcohol. Alcohol-related epigenetic mechanisms include modulation of DNA methylation, histone acetylation/deacetylation, and expression of micro RNA (French 2013). These epigenetic mechanisms associated with alcohol also are known to affect the gastrointestinal-hepatic system (Shukla and Lim 2013) and may promote, for example, the progression of hepatic carcinoma. For the most part, alcohol-related epigenetic changes have not yet been associated with tumor growth, metastasis, and survival; however, alcohol-induced aberrant DNA methylation of certain genes plays a role in the control of breast cancer (Tao et al. 2011). Moreover, alcohol also can dysregulate the immune system through epigenetic mechanisms (Curtis et al. 2013), and this aspect of the association between alcohol, the immune system, and cancer progression needs to be explored further.

Another potential target for future research is a molecule called toll-like receptor 4, which is known to help regulate host innate immunity. This receptor recognizes the lipopolysaccharide (LPS) endotoxin, a molecule found on by certain bacteria that are part of the intestinal microflora. In the blood, LPS can induce strong immune reactions. Alcohol is known to facilitate the release of LPS from the gut into the systemic circulation, and this is a key factor in the pathogenesis of alcoholic liver disease (Petrasek et al. 2010). In addition to its response to LPS, toll-like receptor 4 can facilitate antitumor immune responses; however, emerging evidence also suggests that overactivation of this receptor is associated with tumor progression as well as tumor development (Mai et al. 2013). Although these observations need to be explored further, they suggest that this receptor could be a target for future agonist or antagonist targeted treatment for cancer, particularly for patients that abuse alcohol.

Continued research into the detrimental and beneficial effects of alcohol in human cancer patients and animal models of cancer is a key factor to understanding the complex interactions that affect tumor progression and survival, particularly in the context of alcohol use. This research has a strong potential to discover new immunotherapy and epigenetic approaches to cancer treatment as well as treatment of other alcohol-induced diseases.

## Footnotes

1 In estrogen-positive breast cancer, the cancer cells carry the estrogen receptor and depend on estrogen for growth. In contrast, in triple-negative breast cancer, the cancer cells carry neither estrogen nor progesterone or HER2 receptors.

2 The exception to this is a study in Russia indicating an inverse association between alcohol consumption and mortality (Zaridze et al. 2009).

3 Interestingly, the same cell type was decreased in the peripheral blood in the cancer patients compared with control patients.

4 Epithelial mesenchymal transition is a process whereby epithelial cells lose their innate cellular polarity and cell–cell adhesive properties to become mesenchymal cells, which lack polarity and have the ability to migrate and to invade through tissues.

5 The food included blocks of a jelly-like material (i.e., agar-agar) containing 40 percent alcohol and 0.5 g/kg peanut butter.

6 Epigenetic mechanisms are processes that alter the expression of certain genes without permanently altering the DNA building blocks (i.e., nucleotides) making up the genes; examples of epigenetic mechanisms include the temporary chemical modification (e.g., methylation or acetylation) of nucleotides or of the proteins (i.e., histones) around which the DNA is wrapped in the cell nucleus.
