# Supplementary material for: Effects of Alcohol on Tumor Growth, Metastasis, Immune Response, and Host Survival
Source: Alcohol Res. 2015;37(2):311–22. doi: 10.35946/arcr.v37.2.14 (PMC4590626; doi:10.35946/arcr.v37.2.14)
Supplement: Related texts

# Research article: Effects of Alcohol on Tumor Growth, Invasion, Metastasis, and Survival in Animal Models

**Published:** 2015

Several studies using animal cancer models indicate tumor-specific differences in the effect of alcohol on tumor growth and metastasis. These models included various types of breast cancer, melanoma, lung cancer, colon cancer, and liver cancer (i.e., hepatocellular carcinoma).

## Lung Cancer

One early study (Capel et al. 1978) investigated the effect of alcohol exposure on the growth and metastasis of Lewis lung carcinoma. Male animals from a type of mouse strain called C57BL/6 were exposed to 10 percent ethanol in their drinking water for 2, 4, 5, or 8 weeks before tumor cells were implanted into their thighs. Ethanol administration then was continued for 2 more weeks. The study found that ethanol exposure before tumor injection did not affect tumor growth. Furthermore, metastases were significantly reduced in the 2-week and 8-week ethanol groups but not in the 4-week and 5-week groups. Administration of ethanol for 2 weeks after tumor inoculation affected neither tumor growth nor metastasis. These investigators also evaluated the effect of 2-week pre- and postimplantation ethanol exposure (10 percent in drinking water) on the growth of another type of tumor called Ehrlich ascites carcinoma, which is a spontaneous murine mammary adenocarcinoma adapted to grow in fluid in the abdominal cavity (i.e., ascites). Exposure to ethanol before but not after tumor injection significantly decreased the tumor cell number.

## Colon Cancer

Gu and colleagues (2005) assessed the effects of alcohol on human HT1080 colon cancer cells in a chick embryo model, focusing on variables related to the blood supply of the tumor. One of the variables analyzed was the expression of vascular endothelial growth factor (VEGF)—a growth factor that promotes blood vessel formation (i.e., is proangiogenic) and enhances tumor vascularization. The study found that exposure to 0.25 g/kg ethanol per day for 9 days resulted in a 2.2-fold increase in tumor volume as well as a 3.9-fold increase in the expression of VEGF mRNA in the tumor cells and a 2.1-fold increase in the amount of the blood vessels in the tumor (i.e., intratumoral vascular volume) compared with control embryos. Exposure of isolated tumor cells to 10 mM and 20 mM ethanol for 19 hours also increased VEGF mRNA and protein expression. The increased intratumoral vascular volume strongly correlated with the increase in tumor volume as well as with intratumoral connective tissue volume density. Finally, invasion of HT1080 cells from the tumor into blood vessels (i.e., intravasation), which occurs during metastasis, increased more than eightfold in response to ethanol. Ethanol did not have a direct effect on cell proliferation.

## Hepatocellular Carcinoma

Because excessive alcohol consumption often is associated with liver disease, investigators also have examined the effects of alcohol on hepatocellular carcinoma. One study (Thompson et al. 2013) evaluated the effects of alcohol as well as a high-fat diet on hepatocellular carcinoma progression in male C57BL/6 mice with diet-induced obesity as well as in nonobese control mice. Both groups were injected with Hepa1-6 tumor cells. Ethanol was administered in drinking water at daily alternating concentrations of 10 percent and 20 percent for 6 weeks before the animals were injected with the tumor cells. The alcohol and dietary regimens then were continued for an additional 8 weeks. Gross numbers of tumors as well as tumor burden were lower in mice fed the high-fat diet with and without ethanol or the control diet with ethanol than in control animals receiving neither ethanol nor high-fat diet, with ethanol exerting a greater protective effect than the high-fat diet. However, the high-fat diet both alone and in conjunction with ethanol enhanced mortality in the tumor-bearing mice. Further analyses found that all tumor-bearing mice exhibited increased numbers of inflammatory white blood cells in their livers, regardless of whether they had received ethanol. The expression of some cytokines and their receptors also was altered in the tumor-bearing mice, and these changes in some cases depended on the animals’ diet. Thus, tumor necrosis factor (TNF)-α and interleukin (IL)-6 receptor alpha mRNA expression were increased and transforming growth factor (TGF)-β mRNA expression was decreased in tumor-bearing mice receiving the high-fat diet compared with mice not injected with tumor. An analysis of seven cytokines (IL-1β, IL-6, IL-10, IL-12, IL-13, interferon gamma [IFN-γ], and TNF-α) in the pooled plasmas from each experimental group indicated no changes across the groups except in the water-drinking, high-fat diet group, which exhibited depressed cytokine levels. There was no correlation with ethanol consumption compared with mice not injected with tumor cells.

## Breast Cancer

The effect of ethanol on mammary cancer growth has been studied in a number of animal models, using both rodent and human tumor cell lines.

### Studies Using Rodent Tumor Cell Lines

Wang and colleagues (2012) examined the effect of ethanol on the growth of the aggressive estrogen receptor–positive E0771 mouse mammary cancer in female C57BL/6 mice. The mice were given 2 percent ethanol in drinking water for half a day on each of 3 consecutive days before the E0771 tumor cells were inoculated into breast tissue (i.e., secondary mammary fat pad), and the ethanol feeding regimen then was continued for 24 days. The study found that the ethanol group exhibited higher primary tumor growth rates, increased final tumor weights, and a twofold increase in lung metastases compared with the water-drinking control group. Immunohistochemical analyses of the mammary tumor tissues also showed a higher density of tiny blood vessels in the ethanol group, indicating that ethanol promoted tumor angiogenesis. Finally, the investigators found increased expression of a chemokine called monocyte chemoattractant protein-1, or MCP-1 (also known as CCL2), which has been implicated in tumor development and angiogenesis, in tumor tissues from the ethanol group and in E0771 cells exposed to 0.2 percent ethanol. MCP-1 plays an important role in suppressing antitumor immune functions and facilitating tumor metastasis (Kudo-Saito et al. 2013), indicating another mechanism through which alcohol could promote breast cancer progression.

A related study using the same alcohol-feeding regimen confirmed alcohol’s effects on growth and angiogenesis of E0771 inoculated into other female C57BL/6 mice (Lu et al. 2014). In that study, a molecule that can inhibit VEGF receptor 2 blocked alcohol’s stimulatory effect on tumor growth, indicating that alcohol acts via a VEGF pathway.

Holcomb and colleagues (2012) examined the effects of various diets and supplements on the growth of estrogen receptor–positive mammary tumor cells (derived from mammary tumor virus-Wnt-1 transgenic mice) inoculated subcutaneously into female C57BL/6J mice that had their ovaries removed. Conditions tested included (1) 20 percent weight per volume (w/v) alcohol in drinking water; (2) a calorie-restricted diet with 30 percent fewer calories than normal; (3) a high-fat diet where 60 percent of calories were derived from fat; (4) a low-fat diet where 10 percent of calories were derived from fat; and (5) estrogen supplementation. The diets and alcohol were started when the animals were 8 weeks old and continued for 27 weeks. Estrogen pellets were implanted after 19 weeks of alcohol consumption, and tumors were implanted after 22 weeks. The results on tumor growth were similar to those obtained by Hong and colleagues (2011), with the high-fat diet and alcohol promoting tumor growth and estrogen suppressing it. Tumor growth was greatly inhibited in the mice receiving a high-fat diet as well as estrogen supplements. The calorie-restricted diet also inhibited tumor growth independent of the effects of alcohol and estrogen supplementation. Neither the diets nor alcohol affected VEGF.

Researchers also studied the effects of alcohol on estrogen receptor–negative mouse mammary tumors. One study involving estrogen receptor–negative Met-1 cancer cells used female FVB/N mice that consumed 20 percent w/v ethanol in drinking water for 18 weeks before they were injected subcutaneously with the cancer cells (Hong et al. 2010). Compared with water-drinking control mice, the ethanol-drinking animals developed palpable tumors earlier and also developed larger tumors. Several other parameters (i.e., insulin sensitivity, leptin levels in the blood, and estrogen levels) were elevated in the alcohol-consuming mice. These researchers also examined the effect of ethanol in vitro on the migration of the estrogen receptor–positive T47D breast cancer cell line. The results showed that cells exposed to different concentrations of ethanol from 0.1 percent to 0.5 percent exhibited increased migration, as did cells exposed to estrogen (20 nM). The combination of estrogen and 0.5 percent resulted in higher migration than either treatment alone.

In another study (Hong et al. 2011), the researchers examined the effects of alcohol on Met-1 tumor growth as a function of a diet (i.e., low fat vs. high fat) and estrogen supplementation (low dose vs. high dose). Mice consuming the high-fat diet developed larger tumors than did mice fed the low-fat diet; moreover, alcohol ingestion increased the final tumor volume in both dietary groups. Estrogen treatment suppressed tumor growth regardless of diet and alcohol consumption; however, mice treated with high-dose estrogen had slightly larger tumors than did mice treated with the low dose. Tumor tissues also were analyzed for the levels of various regulatory molecules hypothesized to potentially influence tumor progression, including phosphoinositide 3 kinase (PI3K/p85α); a protein kinase called Akt; the phosphorylated form of Akt (p-AktSer^473^), a signaling molecule associated with cell proliferation, survival, and invasion; proliferating cell nuclear antigen (PCNA); and cleaved caspase-3, a molecule involved in programmed cell death (i.e., apoptosis), with the following results:

- PI3K (p85α) was not affected by alcohol, high-fat diet, or estrogen treatment.
- Alcohol and the high-fat diet increased expression of p-AktSer^473^. In contrast, estrogen supplementation reduced phosphorylation of Akt in both the alcohol and high-fat diet groups.
- The high-fat diet and alcohol decreased the level of cleaved caspase-3, suggesting decreased apoptosis in the tumor tissue. Interestingly, estrogen reduced this signaling molecule in tumors from mice fed alcohol and high-fat diets.
- PCNA was not altered by any of the variables, indicating that cell proliferation was not affected.

Other researchers investigated the effects of alcohol on metastasis of the estrogen receptor–negative and natural killer (NK) cell-sensitive rat MADB106 mammary adenocarcinoma (Yirmiya et al. 1992). In this study, male Fischer 344 rats were administered only one alcohol dose (1.5 to 3.5 g ethanol/kg body weight into the peritoneal cavity) 1 hour before intravenous tumor inoculation. The higher ethanol doses (i.e., 2.5 g/kg and 3.5 g/kg) significantly increased the number of lung metastases, whereas the lowest dose (1.5 g/kg) did not. Administration of naltrexone, an opioid receptor antagonist used to treat alcohol dependence, did not modify the alcohol-related increase in metastasis. Ethanol’s effects on lung metastasis seem to depend on the exact administration schedule. Thus, in a related study these researchers found that administration of 2.5 g/kg ethanol 24 hours before or after tumor inoculation did not affect lung metastasis (Ben-Eliyahu et al. 1996). Yirmiya and colleagues (1992) also administered ethanol in a liquid diet for 2 weeks before and 3 weeks after tumor inoculation and found that lung metastases were increased.

Alcohol’s effects also seem to depend on the tumor type studied. When rats were injected intravenously with rat CRNK-16 leukemia cells instead of MADB106 cells, acute administration of 1.5 to 3.5 g of ethanol/kg/body weight reduced survival in a dose-related fashion, whereas maintenance on a liquid diet containing 5 percent w/v ethanol did not (Ben-Eliyahu et al. 1996; Yirmiya et al. 1992).

One of the ways in which the body defends itself against tumor cells involves their destruction by NK cells. The investigators also analyzed alcohol’s effects on NK-cell activity, finding that neither acute injection nor dietary administration of ethanol in these experiments affected NK-cell activity against MADB106 cells when determined in an in vitro assay (Yirmiya et al. 1992). When MADB106 and CRNK-16 cells were incubated with ethanol in vitro, the numbers of these cells were reduced after 5 days. These effects were significant for MADB106 cells at ethanol concentrations of 0.2 percent, 0.5 percent, and 1.0 percent ethanol and for CRNK-16 cells at 0.5 percent and 1.0 percent ethanol. Ethanol had no effect in rats that were depleted of NK cells, and metastasis was not affected after injection of the NK-insensitive C4047 rat colon cancer cell line (Ben-Eliyahu et al. 1996).

Tumor growth and metastasis also were studied in the highly metastatic estrogen receptor–negative 4T1.2 cells implanted into the mammary fat pad of female Balb/c that had consumed alcohol in drinking water for 4 weeks (Vorderstrasse et al. 2012). The analyses indicated that continuous availability of 1 percent, 5 percent, and 18 percent ethanol w/v did not affect either the establishment of tumors or the final mammary tumor weight, indicating a lack of effect on tumor growth. However, the animals receiving 18 percent ethanol exhibited a marked reduction in metastasis to secondary mammary glands and to the lung. Tumor-associated increases in spleen size (i.e., splenomegaly) also were significantly reduced in the 18-percent ethanol group. Lung metastasis tended to be lower in mice consuming 5 percent ethanol, but there was no effect among those consuming 1 percent ethanol. Although the animals receiving 18 percent ethanol showed suppressed metastasis, they were less healthy compared with the other treatment and control groups as indicated by appearance, body condition, natural behavior, and provoked behavior. In vitro studies indicated that exposure of cells to 0.3 percent w/v ethanol did not affect 4T1.2 cell proliferation, colony formation, or invasion but did reduce cell migration twofold. Reduced cell migration may be related to changes in expression of CXCR4, a chemokine receptor that is important to migration and metastasis of breast and other cancers (Sarvaiya et al. 2013). Vorderstrasse and colleagues (2012) showed that expression of CXCR4 was suppressed 60 to 80 percent in tumors of mice consuming 18 percent ethanol; however, a correlation analysis indicated a lack of relationship between CXCR4 expression and metastasis. When the 4T1.2 cells were grown in 0.3 percent ethanol, however, CXCR4 expression was not altered, suggesting that expression was indirectly modulated by alcohol consumption in vivo.

### Studies Using Human Tumor Cell Lines

Several studies examined the specific effects of ethanol on various aspects of disease progression in human breast cancer cell lines, including proliferation of cells. Singletary and colleagues (2001) found that incubation in 0.4 percent w/v ethanol increased cell proliferation in the estrogen receptor–positive MCF-7 and ZR75.1 breast cancer cells but not in the estrogen receptor–negative BT-20 and MDA-MB-231 cells. The effect of ethanol on MCF-7 cells also was correlated with increases in estrogen receptor alpha content. However, alcohol’s effects are complex. When the MCF-7 cells were cultured together with human skin fibroblasts in 0.4 percent ethanol for 72 hours, ethanol suppressed estrogen receptor alpha expression compared with untreated cells (Sanchez-Alvarez et al. 2013). Thus, the tumor micro-environment is important in determining estrogen-receptor status and the effects of alcohol on breast cancer. Whether this study is relevant to patients with breast cancer is not known. However, additional studies are warranted, because estrogen receptor–negative breast cancer generally is more aggressive, and patients have a worse prognosis than patients with estrogen receptor–positive breast cancer. Moreover, conversion from estrogen receptor alpha positive to estrogen receptor alpha negative can occur (Hoefnagel et al. 2010).

Other studies focused more on the invasion and migration in vitro of estrogen receptor–positive and estrogen receptor–negative human breast cancer cells. One study (Ma et al. 2003) compared the effects of incubation in 0.4 percent w/v ethanol for 48 hours on various breast cancer cell lines. This treatment increased invasion of the estrogen receptor–positive MCF-7 and T47D breast cancer cells as well as the estrogen receptor–negative HS578T, MDA-MB231, and MDA-MB435 cells. Similarly, incubation for 48 hours in 0.1 percent and 0.2 percent w/v ethanol stimulated invasion of estrogen receptor–negative SKBR3 and estrogen receptor–positive BT474 breast cancer cells. In contrast, ethanol exposure did not affect invasion of HB2, an immortalized normal human breast tissue cell line, or estrogen receptor–negative BT20 breast cancer cells. The effects of ethanol may depend not only on the specific cell line examined but also on the ethanol concentration used. Thus, studies from another laboratory demonstrated that exposure to 0.1 percent, 0.2 percent, and 0.5 percent w/v ethanol enhanced invasion of T47D, MCF-7, and MDA-MB231 cells in a dose-dependent manner (Wong et al. 2011). Similarly, Aye and colleagues (2004) examined the effects of exposure for 48 hours to different ethanol concentrations on estrogen receptor–negative SKBR3 and estrogen receptor–positive BT474 breast cancer cells. For both SKBR3 and BT474 cells exposure to 0.1 percent and 0.2 percent w/v ethanol stimulated invasion. A higher dose of 0.4 percent w/v ethanol, however, inhibited invasion of SKBR3 cells and created mixed results for BT474, with one study (Aye et al. 2004) detecting no effect on invasion and another study (Xu et al. 2010) detecting increased invasion.

Invasive ability generally was related to the expression of ErbB2/neu, an epidermal growth factor (EGF) receptor that is amplified in 20 to 30 percent of breast cancer patients, with higher ErbB2/neu levels indicating higher risk of lymph node metastasis and poor prognosis. More detailed studies of the relationship between alcohol, ErbB2/neu, and invasion in the human breast cancer cell line T47D found that activation of the EGF receptor by addition of EGF did not significantly affect ethanol’s ability to enhance invasiveness (Luo and Miller 2000). Conversely, prevention of ErbB2/neu production inhibited the ability of ethanol to increase migration (Luo and Miller 2000).

Numerous studies have sought to identify the mechanisms through which ethanol affects growth and migration of breast cancer cells or the molecules that mediate these effects. The results of such investigations include the following:

- Studies in MDA-MD231 and T47D cells determined that ethanol induced altered adhesion to fibronectin, increased development of lamellipodia,^1^ increased phosphorylation of a protein called focal adhesion kinase, and increased production of several proteins (e.g., ribosomal protein L7a, smooth muscle myosin alkali light chain), all of which can promote cell migration (Xu et al. 2010; Zhu et al. 2001).
- Exposure of T47D cells to 0.5 percent w/v ethanol decreased mRNA expression of *NM23*, a known metastasis suppressor gene (Wong et al. 2011). However, gene expression levels of two other metastasis suppressors, *KISS1* and *MKK4*, were increased by ethanol. Ethanol treatment also modulated the expression of more than 80 other genes associated with regulation of the extracellular matrix and cell adhesion. For example, ethanol induced expression of the gene encoding fibronectin receptor subunit integrin alpha 5 (ITGA5). This receptor activates signaling pathways supporting invasion, and reduced expression of ITGA5 significantly inhibits invasion. The ethanol-induced expression of this gene was blocked by overexpression of the *NM23* gene, indicating an important relationship between these two genes in controlling invasion of T47D breast cancer cells.
- Studies also assessed alcohol’s interactions with enzymes called matrix metalloproteinases (MMPs) such as MMP-2 and MMP-9 that are involved in the breakdown of the extracellular matrix in normal physiological processes as well as in disease processes, such as metastasis. Studies to date indicate no interactions between alcohol and MMPs (Aye et al. 2004; Ranuncolo et al. 2002).

## Melanoma

In one of the first experiments conducted in melanoma, 6- to 8-week-old female CDBA/2F1 mice consumed water or 20 percent alcohol for 52 weeks before being inoculated in a leg with the Cloudman 8-91 melanoma tumor (Ketcham et al. 1963). When the tumors reached a size of 1.5 to 2.0 cm (about 28 days after tumor inoculation), the groups were divided in half, and half of each group had the primary tumor-bearing leg amputated. At 56 days after tumor implantation, the number and size of pulmonary metastases were recorded for all animals. The study detected no substantial or consistent effect of alcohol on the size or incidence of pulmonary metastases. However, surgical removal of the tumor-bearing leg decreased pulmonary metastasis in both ethanol-drinking and water-drinking groups.

Other studies in mice assessed the effects of acute and chronic alcohol consumption on tumor growth and metastasis using B16 melanoma and its more metastatic variants, B16F10 and B16BL6. An early study (Capel et al. 1978) found that mice given 10 percent ethanol in drinking water for 2 weeks before inoculation with B16 melanoma into the thigh showed no altered tumor growth or metastasis compared with water-drinking controls. In another study (Tan et al. 2007), tumor growth and angiogenesis were examined in C57BL/6 mice implanted subcutaneously with B16F10 melanoma cells. The mice had access to regular drinking water and to 1 percent ethanol in their drinking water for 12 hours each per day for 4 weeks, with tumor cells being implanted during the second week of ethanol administration. Compared with animals who only drank water, those who had access to ethanol developed palpable tumors sooner and had 2.2 times greater tumor weights at the end of the study. Analysis of the tumors indicated an increase in VEGF mRNA and VEGF protein, as well as increased tumor angiogenesis. Moreover, another marker of angiogenesis, VEGF-R1 (Flt-1), also was found in a greater number of tumor cells and endothelial cells in the surrounding tissue from the ethanol group compared with the control group.

Wu and Pruett (1999) determined the effects of acute ethanol administration (5 or 6 g/kg body weight) given through a tube inserted into the mouth (i.e., by oral gavage) on melanoma metastasis. Ethanol administered to female B6C3F1 mice 1 hour before intravenous inoculation of B16F10 melanoma cells greatly increased lung metastasis. The number of metastases also was increased when administration of 6 g/kg ethanol occurred 4 or 10 hours after tumor inoculation but not when it occurred 6 hours before tumor inoculation. About half of the increase in lung tumor nodules seemed to be related to ethanol-induced corticosterone production in the adrenal glands. Experiments analyzing whether ethanol-related inhibition of some aspect of NK-cell function also might contribute to the increased metastasis produced found no indication of significantly reduced NK-cell numbers or activity. However, further studies directed at determining the role of NK cells in the acute ethanol-induced increase in melanoma lung metastasis showed that ethanol inhibited experimental induction of NK-cell activity (in response to poly I:C), resulting in approximately a twofold increase in lung tumor nodules compared with control mice (Wu and Pruett 1999). Additional mechanistic studies indicated that a single dose of ethanol increased corticosterone levels (Collier et al. 2000) and also suppressed experimental induction of mRNA for a variety of cytokines (i.e., interferon alpha [IFN-α], interferon beta [IFN-β], IFN-γ, IL-6, IL-12, and IL-15) (Pruett et al. 2003). As a result, concentrations of IFN-α and IL-12 in the blood were decreased. Conversely, ethanol greatly enhanced the levels of IL-10 in the blood. However, these changes in serum cytokines were not mediated by corticosterone. Overall, these studies suggest that the increase in melanoma lung nodule formation after acute ethanol administration is complex and related to effects associated with some aspect of NK-cell inhibition, increased suppression of NK-cell activity by corticosterone, and corticosterone-independent effects on cytokines favoring tumor immune escape.

The effects of chronic alcohol consumption on tumor growth and metastasis of the highly invasive and spontaneously metastatic B16BL6 melanoma inoculated subcutaneously were studied in female C57BL/6 mice administered ethanol in drinking water. In an initial study, consumption of 2.5 percent, 10 percent, or 20 percent w/v ethanol in drinking water for 6 to 8 weeks before tumor inoculation and continuing thereafter did not affect primary tumor growth (Blank and Meadows 1996). However, the animals receiving 20 percent ethanol in their drinking water exhibited consistently reduced survival, lower tumor weight, and lower final body weight compared with the other groups. Alcohol exposure also affected metastasis. All three ethanol-exposed groups had reduced metastasis to the axillary lymph nodes, with the 10-percent and 20-percent ethanol groups showing reduced lung metastasis, and the 20-percent ethanol group showing reduced superficial metastasis to the kidneys. Metastasis did occur, however, in the draining inguinal lymph nodes in mice consuming 20 percent weight per volume ethanol for 12 weeks (Zhang et al. 2011*b*).

More specific experiments sought to determine the effect in mice on metastasis of B16BL6 melanoma of short-term and long-term exposure to 10 percent and 20 percent w/v ethanol before and after intravenous inoculation into a tail vein or inoculation into the skin of the pinna of the ear^2^ (Meadows et al. 1993*a*,*b*; Spitzer et al. 2000; Zhang et al. 2011*a*). These experiments showed that 10 percent w/v ethanol did not affect metastasis after intravenous tumor inoculation in female C57BL/6 mice consuming alcohol for 2 weeks or spontaneous metastasis in mice injected 1 week after initiating ethanol feeding. However, lung metastasis was inhibited if intravenous injection of tumor cells occurred at 4, 6.5, 7, and 12 weeks after initiation of 20 percent w/v ethanol. Similarly, spontaneous metastasis to the lung was significantly inhibited in mice injected with melanoma at 1, 4, 6.5, and 10 weeks of consuming 20 percent ethanol. Ethanol did not prevent spontaneous metastasis to the draining cervical lymph nodes.

The antimetastatic effect of ethanol on the B16BL6 melanoma tumor was confirmed after injection of tumor cells in male C57BL/6J mice that carry a gene for obesity and which had consumed 20 percent ethanol in drinking water for 10 weeks (Kushiro and Nunez 2012). Different groups were analyzed for lung tumor metastasis over a period of 16 to 21 days. All of the water- drinking animals had developed visible lung metastases at 16 days after tumor injection, whereas some of the ethanol-drinking mice did not develop lung metastases until 21 days. More-over, the numbers of lung metastases in the ethanol-drinking mice were significantly lower at 21 days.

The contribution of NK cells to the inhibition of metastasis was evaluated in mice consuming 10 percent or 20 percent w/v ethanol for 4 weeks (Meadows et al. 1993*a*). Whereas consumption of 10 percent ethanol did not alter the NK cells’ ability to destroy other cells (i.e., decrease cytolytic activity), animals consuming 20 percent ethanol showed decreased NK cytolytic activity. And although experimental stimulation of NK cells could enhance their cytolytic activity 4.3-fold in the ethanol-drinking animals, compared with 2.6-fold in the control animals, overall cytolytic activity still was lower in the ethanol group than in the control group. Treatment of mice with an antibody against NK cells (i.e., anti-NK1.1 antibody) markedly decreased NK-cell cytolytic activity in both water- and ethanol-drinking animals. These same treatments were evaluated in mice injected with B16BL6 melanoma. Experimental stimulation of NK cells decreased the number of lung metastases in the water-drinking and 10-percent ethanol groups, but not in the 20-percent ethanol group. Inactivation of NK cells by administration of anti-NK1.1 antibody significantly increased lung metastases in the water-drinking and 10-percent ethanol groups, but not in the 20-percent ethanol group.

The data suggest that inhibition of NK-cell cytolytic activity in mice consuming 20 percent ethanol does not lead to enhanced metastasis following inoculation of B16BL6 melanoma. This lack of interaction between alcohol consumption and NK-cell cytolytic activity in B16BL6 melanoma lung metastasis was further confirmed in another strain of mice (i.e., beige mice) that naturally have low NK cytolytic activity (Spitzer et al. 2000). In other experiments to determine how ethanol decreases metastasis of B16BL6 melanoma, either isolated tumor cells grown in the presence of 0.3 percent ethanol or tumor cells from alcohol-consuming mice were inoculated into water-drinking mice (Blank and Meadows 1996). Mice that had received either tumor cells cultured in the presence of ethanol or derived from mice drinking 20 percent alcohol showed increased lung metastasis compared with control mice or those receiving tumor cells derived from mice drinking 10 percent ethanol. Thus, ethanol seems to increase the actual metastatic potential of melanoma cells. However, because alcohol drinking inhibits metastasis, there seem to be other factors induced by ethanol that counter this metastatic potential.

Further research was directed at identifying factors involved in expression of the antimetastatic effect associated with chronic (12 weeks) consumption of 20 percent w/v alcohol consumption. Zhang and colleagues (2011*a*) found that chronic alcohol consumption increased the numbers of immune cells (e.g., NK, NKT, CD4^+^ T and CD8^+^ T cells) that produce IFN-γ. IFN-γ is an essential factor in the control of melanoma metastasis (Blankenstein and Qin 2003; Ikeda et al. 2002). IFN-γ also mediates the antimetastatic effects of chronic alcohol consumption, because mice that could not produce IFN-γ did not show this effect (Zhang et al. 2011*a*). The investigators attempted to determine whether one immune cell population was more important than another in producing the antimetastatic IFN-γ. However, through a series of specific cell depletion experiments, they found that it did not matter which cell type produced the IFN-γ.

Other investigators (Kushiro and Nunez 2012) found that B16BL6 melanoma cells grown in 0.1 percent, 0.2 percent, or 0.5 percent ethanol showed considerably reduced cell invasion and, at the highest ethanol concentration, reduced cell motility and anchorage-dependent growth. Ethanol did not affect cell proliferation, apoptosis, or necrosis of the melanoma cells. In addition, the highest ethanol dose altered the expression of several genes that play prominent roles in regulating melanoma metastasis (i.e., the IL6, Nfkb, snail1, E-cadherin, Kiss1, Nm23-m1, and Nm23-m2 genes). These changes also could contribute to the antimetastatic effect of alcohol on melanoma.

## Summary

Animal models have yielded some insights into the effects of alcohol on tumor growth, survival, and metastasis of different cancers, including breast cancer, lung cancer, liver cancer, and melanoma. However, because cancer is a collection of many different diseases and subtypes, each cancer or cancer subtype might not respond similarly to alcohol, as is evident from the research discussed here.

The relationship between alcohol and tumor progression is complex. Tumor metastasis involves many steps and also is controlled by many different signaling pathways. Because each cancer is unique, the specific connections between alcohol and the steps involved in cancer progression also by nature are complex. Thus, it is important to relate experimental results to specific types/subtypes of cancer as well as the amount of alcohol consumed, the route of alcohol administration, and the duration of alcohol administration. Clearly, more studies are needed to define the effect of alcohol on tumor progression and to determine the mechanisms associated with individual cancers and subtypes.

## Footnotes

1 Lamellipodia are projections on the mobile edge of a cell that allow the cell to move across a substrate.

2 This method of tumor placement facilitates spontaneous metastasis.

## References

[b110-arcr-37-2-311] Aye MM, Ma C, Lin H (2004). Ethanol-induced in vitro invasion of breast cancer cells: The contribution of MMP-2 by fibroblasts. International Journal of Cancer.

[b111-arcr-37-2-311] Ben-Eliyahu A, Page GG, Yirmiya R, Taylor AN (1996). Acute alcohol intoxication suppresses natural killer cell activity and promotes tumor metastasis. Nature Medicine.

[b112-arcr-37-2-311] Blank SE, Meadows GG (1996). Ethanol modulates metastatic potential of B16BL6 melanoma and host responses. Alcoholism: Clinical and Experimental Research.

[b113-arcr-37-2-311] Blankenstein T, Qin Z (2003). The role of IFN-gamma in tumor transplantation immunity and inhibition of chemical carcinogenesis. Current Opinion in Immunology.

[b114-arcr-37-2-311] Capel ID, Jenner M, Pinnock MH (1978). The effect of chronic ethanol intake on the growth and spread of some murine tumors. Oncology.

[b115-arcr-37-2-311] Collier SD, Wu WJ, Pruett SB (2000). Ethanol suppresses NK cell activation by polyinosinic-polycytidylic acid (poly I:C) in female B6C3F1 mice: Role of endogenous corticosterone. Alcoholism: Clinical and Experimental Research.

[b116-arcr-37-2-311] Gu JW, Bailey AP, Sartin A (2005). Ethanol stimulates tumor progression and expression of vascular endothelial growth factor in chick embryos. Cancer.

[b117-arcr-37-2-311] Hoefnagel LD, van de Vijver MJ, van Slooten HJ (2010). Receptor conversion in distant breast cancer metastases. Breast Cancer Research.

[b118-arcr-37-2-311] Holcomb VB, Hong J, Nunez NP (2012). Exogenous estrogen protects mice from the consequences of obesity and alcohol. Menopause.

[b119-arcr-37-2-311] Hong J, Holcomb VB, Kushiro K, Nunez NP (2011). Estrogen inhibits the effects of obesity and alcohol on mammary tumors and fatty liver. International Journal of Oncology.

[b120-arcr-37-2-311] Hong J, Holcomb VB, Tekle SA (2010). Alcohol consumption promotes mammary tumor growth and insulin sensitivity. Cancer Letters.

[b121-arcr-37-2-311] Ikeda H, Old LJ, Schreiber RD (2002). The roles of IFN gamma in protection against tumor development and cancer immunoediting. Cytokine & Growth Factor Reviews.

[b122-arcr-37-2-311] Ketcham AS, Wexler H, Mantel N (1963). Effects of alcohol in mouse neoplasia. Cancer Research.

[b123-arcr-37-2-311] Kudo-Saito C, Shirako H, Ohike M (2013). CCL2 is critical for immunosuppression to promote cancer metastasis. Clinical & Experimental Metastasis.

[b124-arcr-37-2-311] Kushiro K, Nunez NP (2012). Ethanol inhibits B16-BL6 melanoma metastasis and cell phenotypes associated with metastasis. In Vivo.

[b125-arcr-37-2-311] Lu Y, Ni F, Xu M (2014). Alcohol promotes mammary tumor growth through activation of VEGF-dependent tumor angiogenesis. Oncology Letters.

[b126-arcr-37-2-311] Luo J, Miller MW (2000). Ethanol enhances erbB-mediated migration of human breast cancer cells in culture. Breast Cancer Research and Treatment.

[b127-arcr-37-2-311] Ma C, Lin H, Leonard SS (2003). Overexpression of ErbB2 enhances ethanol-stimulated intracellular signaling and invasion of human mammary epithelial and breast cancer cells in vitro. Oncogene.

[b128-arcr-37-2-311] Meadows GG, Elstad CA, Blank SE (1993a). Alcohol consumption suppresses metastasis of B16-BL6 melanoma in mice. Clinical & Experimental Metastasis.

[b129-arcr-37-2-311] Meadows GG, Elstad CA, Blank SE (1993b). Ethanol and melanoma metastasis. Advances in the Biosciences Alcohol, Drugs of Abuse, and Immunomodulation (AIDS): Proceedings of the 2nd International Conference on Alcohol, Drugs of Abuse and Immunomodulation (AIDS) Held in Arizona, USA, on 9–13 September 1992.

[b130-arcr-37-2-311] Pruett SB, Fan R, Zheng Q (2003). Acute ethanol administration profoundly alters poly I:C-induced cytokine expression in mice by a mechanism that is not dependent on corticosterone. Life Sciences.

[b131-arcr-37-2-311] Ranuncolo SM, Matos E, Loria D (2002). Circulating 92-kilodalton matrix metalloproteinase (MMP-9) activity is enhanced in the euglobulin plasma fraction of head and neck squamous cell carcinoma. Cancer.

[b132-arcr-37-2-311] Sanchez-Alvarez R, Martinez-Outschoorn UE, Lin Z (2013). Ethanol exposure induces the cancer-associated fibroblast phenotype and lethal tumor metabolism: Implications for breast cancer prevention. Cell Cycle.

[b133-arcr-37-2-311] Sarvaiya PJ, Guo D, Ulasov I (2013). Chemokines in tumor progression and metastasis. Oncotarget.

[b134-arcr-37-2-311] Singletary KW, Frey RS, Yan W (2001). Effect of ethanol on proliferation and estrogen receptor-alpha expression in human breast cancer cells. Cancer Letters.

[b135-arcr-37-2-311] Spitzer JH, Nunez NP, Meadows SA (2000). The modulation of B16BL6 melanoma metastasis is not directly mediated by cytolytic activity of natural killer cells in alcohol-consuming mice. Alcoholism: Clinical and Experimental Research.

[b136-arcr-37-2-311] Tan W, Bailey AP, Shparago M (2007). Chronic alcohol consumption stimulates VEGF expression, tumor angiogenesis and progression of melanoma in mice. Cancer Biology & Therapy.

[b137-arcr-37-2-311] Thompson KJ, Swan RZ, Iannitti DA (2013). Diet-induced obesity and ethanol impair progression of hepatocellular carcinoma in a mouse mesenteric vein injection model. Surgical Endoscopy.

[b138-arcr-37-2-311] Vorderstrasse BA, Wang T, Myers AK (2012). Alcohol consumption suppresses mammary tumor metastasis in a syngeneic tumor transplantation model. Breast Cancer Research and Treatment.

[b139-arcr-37-2-311] Wang S, Xu M, Li F (2012). Ethanol promotes mammary tumor growth and angiogenesis: The involvement of chemoattractant factor MCP-1. Breast Cancer Research and Treatment.

[b140-arcr-37-2-311] Wong AW, Paulson QX, Hong J (2011). Alcohol promotes breast cancer cell invasion by regulating the Nm23-ITGA5 pathway. Journal of Experimental & Clinical Cancer Research.

[b141-arcr-37-2-311] Wu WJ, Pruett SB (1999). Ethanol decreases host resistance to pulmonary metastases in a mouse model: Role of natural killer cells and the ethanol-induced stress response. International Journal of Cancer.

[b142-arcr-37-2-311] Xu M, Bower KA, Wang S (2010). Cyanidin-3-glucoside inhibits ethanol-induced invasion of breast cancer cells overexpressing ErbB2. Molecular Cancer.

[b143-arcr-37-2-311] Yirmiya R, Ben-Eliyahu S, Gale RP (1992). Ethanol increases tumor progression in rats: Possible involvement of natural killer cells. Brain, Behavior, and Immunity.

[b144-arcr-37-2-311] Zhang H, Zhu Z, McKinley JM, Meadows GG (2011a). IFN-gamma is essential for the inhibition of B16BL6 melanoma lung metastasis in chronic alcohol drinking mice. Clinical & Experimental Metastasis.

[b145-arcr-37-2-311] Zhang H, Zhu Z, Meadows GG (2011b). Chronic alcohol consumption decreases the percentage and number of NK cells in the peripheral lymph nodes and exacerbates B16BL6 melanoma metastasis into the draining lymph nodes. Cell Immunology.

[b146-arcr-37-2-311] Zhu Y, Lin H, Li Z (2001). Modulation of expression of ribosomal protein L7a (rpL7a) by ethanol in human breast cancer cells. Breast Cancer Research and Treatment.
